# Examining the incremental impact of long-standing health conditions on subjective well-being alongside the EQ-5D

**DOI:** 10.1186/1477-7525-12-61

**Published:** 2014-04-29

**Authors:** Mengjun Wu, John Brazier, Clare Relton, Cindy Cooper, Christine Smith, Joanna Blackburn

**Affiliations:** 1 Institute of Mental Health, University of Nottingham, Innovation Park, Jubilee Campus, Triumph Road, Nottingham NG7 2TU, UK; 2 School of Health and Related Research, University of Sheffield, Regent Court, 30 Regent Street, Sheffield S1 4DA, UK; 3 Research and Development, Barnsley Hospital NFT, Gawber Road, Barnsley S75 2EP, UK

**Keywords:** EQ-5D, Long-standing health conditions, Subjective well-being, Life satisfaction

## Abstract

**Background:**

Generic preference-based measures such as the EQ-5D and SF-6D have been criticised for being narrowly focused on a sub-set of dimensions of health. Our study aims to explore whether long-standing health conditions have an incremental impact on subjective well-being alongside the EQ-5D.

**Methods:**

Using data from the South Yorkshire Cohort study (N = 13,591) collected between 2010 and 2012 on the EQ-5D, long-standing health conditions (self-reported), and subjective well-being measure – life satisfaction using a response scale from 0 (completely dissatisfied) to 10 (completely satisfied), we employed generalised logit regression models. We assessed the impact of EQ-5D and long-standing health conditions together on life satisfaction by examining the size and significance of their estimated odds ratios.

**Results:**

The EQ-5D had a significant association with life satisfaction, in which anxiety/depression and then self-care had the largest weights. Some long-standing health conditions were significant in some models, but most did not have an independent impact on life satisfaction. Overall, none of the health conditions had a consistent impact on life satisfaction alongside the EQ-5D.

**Conclusions:**

Out study suggests that the impact of long-standing health conditions on life satisfaction is adequately captured by the EQ-5D, although the findings are limited by reliance on self-reported conditions and a single item life satisfaction measure.

## Background

Generic preference-based measures (GPBMs) such as the EQ-5D and SF-6D are commonly used to obtain health utility scores for the calculation of Quality Adjusted Life Years in the economic evaluation of health technologies and interventions to inform decisions [1-3]. While generic measures are intended for all patient population, it is not possible for them to cover all aspects of health. There are 5 to 8 dimensions in the commonly used GPBMs such as the EQ-5D [4], SF-6D [5], and HUI3 [6,7], which cover the main aspects of health. The EQ-5D has dimensions for mobility, self-care, usual activities, pain/discomfort, and anxiety/depression. There is a concern that these dimensions may not capture all the important impacts of health conditions on people’s lives [8]. This paper aims to examine this concern by estimating the incremental impact of long-standing health conditions on subjective well-being (SWB) alongside the EQ-5D.

SWB is an umbrella term for how people think and feel about their lives that usually combines hedonic and satisfaction accounts. It is often presented as comprising of three elements “people's longer-term levels of pleasant affect, lack of unpleasant affect, and life satisfaction." [9]: 103. Athaud-Day and colleagues [10] tested the construct validity of SWB by looking at responses to a range of evaluative and affect-based scales. They found a three factor solution which is seen as supporting the ‘tripartite’ theory of SWB of positive affect, negative affect and life satisfaction [10]: 465. Life satisfaction can be overall aspects of life or be concerned with specific aspects of life such a health, work and family.

SWB provides a method for examining the extent that a health measure like the EQ-5D is able to reflect all those things important to someone with a long-standing health condition. When people are asked to provide global assessments of their life or domains of life such as satisfaction with life overall and health, SWB is measured as an evaluation [11]. Life satisfaction can be defined as how people evaluate their lives as a whole rather than their current feelings. It reflectively assesses which life circumstances and conditions are important [12]. The concept of life satisfaction has been used by economists for some time because this measure is prevalent in international and national surveys, including the British Household Panel Survey (BHPS), and is comprehensive and appealing to policy makers [13]. For instance, Dolan and colleagues [14] found that life satisfaction was correlated with health, personal characteristics such as age, gender and ethnicity, education, and employment status. The Life Satisfaction with Life used in much of this research was developed by Pavot and Diener [15] to be a measure of overall life satisfaction. It has been shown to have a good convergent validity with other measures of SWB, temporal stability and sensitivity to change during clinical interventions [15].

Previous studies in the health economics literature have examined the way that SWB might be used to value health described using GPBMs such as the EQ-5D and SF-6D. For instance, data (N = 14,000) from Latin American countries were employed to assess the relationship between the EQ-5D and both health and life satisfaction. The findings were that anxiety and pain tended to be more strongly associated with SWB than physical health, and had larger effects on health satisfaction than on life satisfaction [16]. The BHPS (N = 10,000) and US data (N = 1,173) for example, were used to explore the relationship between the SF-6D and both the day effect and life satisfaction, mental health tended to have the largest negative impact, followed by pain [16-18]. Similar findings about mental health and pain were reported when assessing the relationship between happiness and both the EQ-5D and SF-6D using a Welsh hospital patient sample (N = 15,184). Furthermore, their results also showed that vitality and social functioning had strong associations with happiness [19].

Our intention is to take this research further and to examine whether any long-standing health condition has additional impact on SWB – life satisfaction over and above the EQ-5D. In this study we used a large UK data set of self-reported questionnaire data containing information on life satisfaction, the EQ-5D and a list of long-standing health conditions to examine the impact of the EQ-5D and long-standing health conditions on life satisfaction.

## Methods

### Data

Data for this study were from the South Yorkshire Cohort (SYC) [20], which is a postal and online patient self-completed Health Questionnaire of patients aged 16 to 85 registered with 42 GP practices in South Yorkshire. The SYC protocol was approved by NHS Research Ethics Committee on 27^th^ April 2010 (09/H1306/97). Each patient received an invitation letter and a Health Questionnaire from their GP practice. This study was based on a single wave data set from completed questionnaires from June 2010 to June 2012 with a response rate of 17.8%. The data set had 18,093 patient observations [20]. Our analysis focused on 13,591 patient observations with non-missing data for the variables chosen for this study.

### Dependent variable

There are intensity and frequency questions regarding life satisfaction, happiness or affect which could be used to measure SWB [21]. For example, in relation to intensity questions, a person could be given a scale of completely happy to completely unhappy to select how happy s/he is. A life satisfaction question (i.e. an intensity question) was used in the SYC, which asks: “Thinking about your own life and personal circumstances, how satisfied are you with your life as a whole?” [22]. There were 11 options available for respondents to choose from completely dissatisfied to completely satisfied on a scale of 0–10, in which 0 represented completely dissatisfied and 10 represented completely satisfied.

### Independent variables

We used one GPBM, the EQ-5D, and self-reported long-standing health conditions. The EQ-5D has five dimensions, which are mobility, self-care, usual activities, pain/discomfort and anxiety/depression. There are three levels within each dimension, namely no problems, moderate problems and serious problems. Thus in total 243 health states are defined [4]. We omitted the first level of the EQ-5D dimensions as the reference category and therefore transformed the three levels into two dummy variables (Additional file 1: Table S1). Furthermore, the second and third levels of mobility were combined since there were a small proportion of individuals reporting serious problems in mobility. The same applied to self-care. Therefore, eight dummy variables in total were derived for the EQ-5D. Since the dummy variables denoted worse health states, we expected to observe a negative relationship with life satisfaction which should be stronger as the level of health state increases. The EQ-5D utility scores ranged from −0.594 to 1.

Participants were asked “Do you have any long-standing illness, health problem, condition or disability? If yes, please tick all that apply”. The list included: pain, insomnia, anxiety/nerves, depression, diabetes, breathing problems (e.g. chronic bronchitis, asthma or emphysema), high blood pressure, heart disease, osteoarthritis, stroke and cancer. We included insomnia, diabetes, breathing problems, high blood pressure, heart disease, osteoarthritis, stroke and cancer to be tested in our models. Pain, anxiety/nerves and depression which are already covered in the EQ-5D were therefore not included (Additional file 1: Table S1).

The SYC data set also contained socio-demographic characteristics, namely age, gender, ethnicity, education, socio-economic status (occupation is used as a proxy for this variable), and current employment status. These were used in our models as control variables, and if any control variable turned out to be insignificant, it would be dropped on the basis of no effect on the size and sign of the coefficients in the rest of the model.

### Analysis

First, we presented descriptive statistics of life satisfaction, the EQ-5D, long-standing health conditions and socio-demographic characteristics of the sample.

Second, we employed regression models to examine the impact of additional input of long-standing health conditions on life satisfaction alongside the EQ-5D. Since life satisfaction was treated as an ordinal variable, we considered using an ordered logit model [23]:

$$y^{*}\;=\;{\mathrm{\beta x}}^{'}+e$$

Where y^*^ is the unobserved dependent variable, life satisfaction, x^'^ is the vector of independent variables of the EQ-5D dimensions, long-standing health conditions and socio-demographic characteristics, β  is the vector of coefficients of independent variables and e is the error term.

However, it is possible to estimate the response probability of choosing different levels of life satisfaction based on cut points [23].

$$P\left(y\;\leq \;J|x^{'}\right)\;=\;\alpha_{J}\;-\;{\mathrm{\beta x}}^{'}$$

where J is 0–10, and α_J_ are threshold parameters which indicate the estimates of cut points on life satisfaction to differentiate respondents choosing from one level to the next.

The coefficients were reported in odds ratios form, and odds ratios have values greater or less than one. When they are greater than one, they indicate the likelihood of choosing higher levels of life satisfaction and vice versa. Therefore, we expected to observe odds ratios less than one for the EQ-5D dimensions and long-standing health conditions. Models were compared using the relative size and significance of individual parameter estimates.

However, the ordered logit model can be only used for data which holds the proportional odds assumption. It is an assumption underlying this model, where the relationship between each pair of outcome groups is statistically the same [23]. In other words, the proportional odds assumption is that the coefficients that describe the relationship between the lowest level of life satisfaction versus all higher levels of life satisfaction are the same as those that describe the relationship between the second lowest category and all higher levels of life satisfaction. Because the relationship between all levels of life satisfaction is the same, there is only one set of coefficients. We used a Brant test to determine whether this assumption holds. If the assumption does not hold, the ordered logit should be replaced with a partial proportional odds ordered logit model namely generalised model. This model would allow the coefficients to vary by the levels of life satisfaction to accommodate those independent variables that violate the assumption [24].

In addition, the life satisfaction scale was rescaled due to small numbers in some categories and to avoid a long list of coefficients for each independent variable using the generalised logit.

## Results

### Descriptive statistics

**Figure 1:**
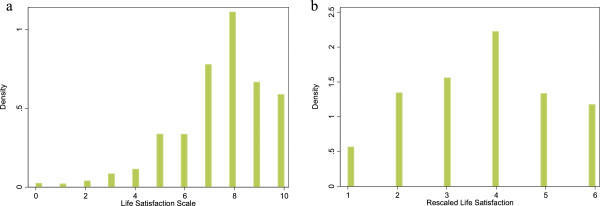
**Distribution of life satisfaction.** Figure 1a shows the distribution of life satisfaction re- sponses over the 10 point life satisfaction scale. The effect of rescaling onto a 1–6 scale are shown in Figure 1b (where scale ranging from 0–4 was grouped as 1, scale ranging from 5–6 was grouped 2, and the remaining scale ranging from 7–10 were rated as 3–6 respectively). The rescaled version provided a relatively normal distribution with a mean life satisfaction at about scale 4 with the largest pro- portion (≈27%) among all categories.

Figure 1a shows the distribution of life satisfaction responses over the 10 point life satisfaction scale. The effect of rescaling onto a 1–6 scale are shown in Figure 1b (where scale ranging from 0–4 was grouped as 1, scale ranging from 5–6 was grouped 2, and the remaining scale ranging from 7–10 were rated as 3–6 respectively). The rescaled version provided a relatively normal distribution with a mean life satisfaction at about scale 4 with the largest proportion (≈27%) among all categories.

The mean age was 53.9 with more of younger people (15–40 and 41–65) reporting higher levels (levels 5 and 6) of life satisfaction than older people (over 65) (i.e. 53% vs. 41%). Males accounted for about 44% of respondents, and there were slightly more males reporting higher levels of life satisfaction than females (i.e. 32% vs. 30%) (Table 1). The mean score (standard deviation – s.d.) of EQ-5D was 0.83 (0.23), which is very close to the UK general population values of 0.85 (0.23) [25]. There were fewer respondents reporting being in full health (1.0) for the EQ-5D compared to the general population (i.e. 47% vs. 52%). There was a similar proportion reporting health being worse than dead compared to the general population (i.e. 2% vs. 1.6%) [25]. Table 2 shows that there were very small proportions reporting being in the worst level in mobility and self-care with 0.1% and 0.2% respectively.

**Table 1:** Sample characteristics

	**N**	**%**
Age distribution		
15-40	3,213	23.6
41-65	6,536	48.1
Over 65	3,842	28.3
Mean age (s.d.)	53.9 (16.8)	
Gender		
Male	5,935	43.7
Female	7,656	56.3
Ethnicity		
White	13,082	96.2
Non-white	509	3.8
Educational attainment		
No qualification	2,733	20.1
GCSEs	2,102	15.5
A levels	4,160	30.6
Degree	4,596	33.8
Socio-economic status		
Blue collar	4,312	31.7
White collar	9,279	68.3
Current employment status		
No	5,868	43.2
Yes	7,723	56.8
How satisfied are you with your life as a whole?		
Completely dissatisfied - completely satisfied		
1	939	6.9
2	2,226	16.4
3	2,583	19.0
4	3,684	27.1
5	2,209	16.2
6	1,950	14.4
Mean self-reported life satisfaction (s.d.)	7.5 (1.9)	
Mean self-reported rescaled life satisfaction (s.d.)	3.7 (1.5)	
Mean EQ-5D score (s.d.)	0.83 (0.23)	
N	13,591	

**Table 2:** Distribution of the EQ-5D dimensions

**N (%)**	**Mobility**	**Self-care**	**Usual activities**	**Pain/discomfort**	**Anxiety/depression**
Level 1	10,745(79.0)	12,826(94.4)	10,913(80.3)	7,933(58.4)	10,194(75.0)
Level 2	2,836(20.9)	735(5.4)	2,488(18.3)	5,141(37.8)	3,145(23.1)
Level 3	10(0.1)	39(0.2)	190(1.4)	517(3.8)	252(1.9)
N	13,591				

Table 3 presents mean EQ-5D scores of all respondents with and without each long-standing health condition.

**Table 3:** Mean EQ-5D scores for respondents with and without long-standing health conditions

**Variables**		**Mean EQ-5D score (s.d.)**	
**Long-standing health conditions**	**Yes – N (%)**	**Yes**	**No**
Insomnia	849(6.3)	0.569(0.331)	0.848 (0.208)
Diabetes	796(5.9)	0.681(0.300)	0.840 (0.220)
Breathing problems	1,360(10.0)	0.695(0.308)	0.846 (0.212)
High blood pressure	2,457(18.1)	0.731(0.277)	0.853 (0.210)
Heart disease	755(5.6)	0.655(0.285)	0.841 (0.220)
Osteoarthritis	1,192(8.8)	0.587(0.288)	0.854 (0.207)
Stroke	239(1.8)	0.617(0.324)	0.835 (0.224)
Cancer	371(2.7)	0.727(0.258)	0.834 (0.227)

The scores did not include comorbidities, instead they were the average values of all respondents who only selected one single health condition. As expected, scores were much lower for respondents with long-standing health conditions ranging from 0.57 to 0.73, where respondents with insomnia had the lowest score and respondents with high blood pressure had the highest score. But compared to respondents with each health condition, respondents without each condition had much higher scores ranging from 0.83 to 0.85. The largest score difference occurred between respondents with insomnia and those without insomnia and the lowest occurred between respondents with high blood pressure and those without high blood pressure.

### Generalised logit model

The proportional odds assumption is violated, so that means that the coefficients that describe the odds ratios between the lowest level of life satisfaction versus all higher levels of life satisfaction are different from those that describe the relationship between the second lowest level of life satisfaction and all higher levels of life satisfaction. Because the relationship between all levels of life satisfaction is different, there are five sets of coefficients presented in Table 4 that reflect the 5 transitions along the 6 point life satisfaction scale. This makes interpretation of the coefficients more complicated since the relative size of the odds ratios may vary across the thresholds.

**Table 4:** Generalised logit regression: EQ-5D and long-standing health conditions

**Life satisfaction level**	**I**		**II**		**III**		**IV**		**V**	
**Independent variables**	**OR**	**SE**	**OR**	**SE**	**OR**	**SE**	**OR**	**SE**	**OR**	**SE**
Mobility 2	0.868	0.107	0.888	0.066	0.911	0.060	0.958	0.069	0.993	0.093
Self-care 2	0.888	0.144	0.785**	0.084	0.827**	0.080	0.781**	0.086	0.755*	0.115
Usual activities 2	1.100	0.138	1.043	0.079	1.111	0.074	1.005	0.074	0.983	0.095
Usual activities 3	0.886	0.232	1.040	0.194	1.083	0.189	0.943	0.192	1.229	0.322
Pain/discomfort 2	1.010	0.086	0.948	0.049	0.911**	0.040	0.914*	0.043	0.939	0.057
Pain/discomfort 3	0.876	0.170	0.945	0.120	0.823*	0.095	0.849	0.112	0.834	0.149
Anxiety/depression 2	0.653***	0.052	0.653***	0.032	0.699***	0.031	0.784***	0.038	0.895*	0.057
Anxiety/depression 3	0.261***	0.044	0.519***	0.072	0.695***	0.094	0.874	0.136	0.884	0.186
Insomnia	0.824	0.101	0.889	0.073	0.894	0.068	0.866	0.076	0.767**	0.094
Diabetes	0.722**	0.095	0.940	0.083	0.984	0.076	1.035	0.086	1.052	0.114
Breathing problems	0.868	0.093	0.894*	0.060	0.905*	0.054	0.968	0.063	0.940	0.081
High blood pressure	1.004	0.093	1.024	0.058	1.027	0.050	1.064	0.055	0.993	0.066
Heart disease	1.205	0.190	1.218**	0.117	1.103	0.090	1.014	0.088	1.019	0.113
Osteoarthritis	0.925	0.115	0.933	0.072	0.985	0.067	1.046	0.077	0.882	0.088
Stroke	0.836	0.197	0.943	0.146	0.799	0.109	0.897	0.135	0.927	0.181
Cancer	1.180	0.255	0.965	0.120	0.965	0.105	0.930	0.111	1.019	0.153
Control variables										
Age	0.944***	0.012	0.963***	0.007	0.959***	0.006	0.951***	0.006	0.947***	0.008
Age^2^	1.001***	0.000	1.000***	0.000	1.001***	0.000	1.001***	0.000	1.001***	0.000
White	1.218	0.195	1.463***	0.145	1.311***	0.121	0.928	0.095	0.608***	0.074
Degree	1.055**	0.026	1.039***	0.015	1.015	0.013	0.980	0.013	0.931***	0.017
Observations	13591									
Likelihood ratio χ^2^	889.60									
P value	0.0000									

Exponentiated coefficients; standard errors in parentheses *p < 0.1, **p < 0.05, ***p < 0.01.

I – Life satisfaction level 1 compared to 2, 3, 4, 5 and 6.

II – Life satisfaction levels 1 and 2 compared to 3, 4, 5 and 6.

III – Life satisfaction levels 1, 2 and 3 compared to 4, 5 and 6.

IV – Life satisfaction levels 1, 2, 3 and 4 compared to 5 and 6.

V – Life satisfaction levels 1, 2, 3, 4 and 5 compared to 6.

Reference categories: no problems in walking about, with self-care & performing usual activities; no pain/discomfort; no anxiety/ depression; no health condition; non-white, below degree.

OR: odds ratio; SE: standard error.

Odds ratios were less than one for all the EQ-5D dimensions apart from usual activities which was more than one in four out of five cases. There were smaller odds ratios for the more severe levels in most cases (i.e. the third level). This indicates that the other four dimensions had a negative impact on life satisfaction with the odds ratios was ranked from highest to lowest (i.e. from least to most impact on life satisfaction) as follows: mobility, pain/discomfort, self-care and anxiety/depression. The odds ratios were statistically significantly less than one at the 5% level for only for anxiety/depression, self-care and pain/discomfort with 4, 3 and 1 respectively. These results indicate that anxiety/depression had the largest negative impact on life satisfaction, followed by self-care. However, this pattern was not consistent across thresholds of life satisfaction since the results indicate that severe anxiety/depression had more impact than self-care problems at lower levels of life satisfaction (columns I-III), but the opposite was the case for higher levels of satisfaction. Overall, respondents having a problem in any of these two dimensions were more likely to report lower levels of life satisfaction compared to having no problem.

For pain/discomfort, the odds ratios were statistically significantly less than one at the 5% level for column III, but not the others. Usual activities had odds ratios greater than one, indicating that respondents with problems in performing usual activities were likely to report higher levels of life satisfaction when controlling for all other dimensions and long-standing health conditions, but the odds ratios were not statistically significant. Similarly, the odds ratios for mobility were not statistically significant.

In terms of long-standing health conditions, the odds ratios were less than one for all long-standing health conditions apart from high blood pressure and heart disease that were greater than one in most cases. The odds ratios were statistically significantly less than one at the 5% level for two of the eight conditions: insomnia and diabetes. The odds ratio for insomnia was statistically significantly less than one at the 5% level in Column V, indicating that respondents with insomnia were likely to be completely dissatisfied with their lives. Diabetes had odds ratio less than one and statistically significant at the 5% level in Column I, indicating that respondents with diabetes were likely to be completely dissatisfied with their lives. Surprisingly, heart disease had odds ratio greater than one in Column II and the odds ratio was statistically significant at the 5% level, indicating that respondents with heart disease were likely to report higher levels of life satisfaction. None of all other health conditions had significant coefficients across the model.

In relation to socio-demographic variables, variables including gender, some types of educational attainment, socio-economic status and current employment status were not statistically significant and thus were dropped from the model. The exclusion of these variables did not affect the size and sign of the coefficients in the rest of the model. Therefore, only the coefficients of age, ethnicity and one type of educational attainment of a degree were reported. Odds ratios were less than one for age but greater than one for age squared and were statistically significant, indicating that age had a negative association with life satisfaction but age squared had a positive correlation with life satisfaction. Ethnicity – white was associated with odds ratios greater than one in Columns II and III compared to less than one in Column V, indicating that the difference between respondents who reported lower levels of life satisfaction and those who reported the highest level was significant. The same applied to educational attainment at a degree level.

## Discussion

We tested the criticism that GPBMs are too narrowly focused on a sub-set of health dimensions by exploring whether aspects of health important to people in terms of the impact on life satisfaction were not being included by the EQ-5D. Overall, we found that long-standing health conditions were not associated with significant decrements in life satisfaction once they were entered into a model alongside the EQ-5D. Whilst most had odds ratios less than one, some actually had ratios of more than one and only two were found to be significant and then only across one of the thresholds.

The results on the impact of EQ-5D on life satisfaction and SWB more generally were broadly consistent with the existing literature [26,16-19] that showed anxiety/depression or mental health has a strong, negative association with SWB (e.g. health satisfaction, life satisfaction, day effect, and happiness). Pain, vitality and social functioning were found to have less effect on SWB and physical health tended to have no effect. Our study showed that anxiety/depression had the largest negative association with life satisfaction among five dimensions, whilst mobility had least, which are same as Mukuria and Brazier’s [19] findings although they used happiness as the SWB measure. There is perhaps one discrepant result in our model. Surprisingly, we observed that self-care tended to have the second largest negative association with life satisfaction among five dimensions. However, Mukuria and Brazier found that self-care, usual activities and pain/discomfort had similar negative impact on happiness. One possible explanation could be that the Health Outcomes Data Repository data set, used by Mukuria and Brazier, was patients with more serious health problems.

In terms of socio-demographic characteristics, we also found results similar to the existing literature [14]. For example, age had a negative association with life satisfaction but age squared had a positive correlation with life satisfaction. Being white and educational attainment at a degree level tended to be positively associated with SWB in the existing literature. However, we observed different results for those respondents who reported lower levels of life satisfaction and those who reported the highest level. White and educational attainment were positively associated with life satisfaction for lower levels of life satisfaction, but this became negative for the highest two thresholds.

In relation to our proposed question on whether any long-standing health condition has additional impact on life satisfaction alongside the EQ-5D, we noticed that none of long-standing health conditions tended to stand out and had a consistent, negative association with life satisfaction. Our results showed that only insomnia and diabetes were significant for any threshold, but in each case only one. These results might suggest that the EQ-5D captures everything that matters to respondents in terms of their life satisfaction, but this conclusion must be viewed with some caution given the limitations of this study.

There are a number of important limitations of this study. The long-standing health conditions were self-reported so may not have been accurate compared to clinical diagnoses. There was no indicator of the severity of the conditions and this may have demonstrated a stronger association with life satisfaction. There was also no indicator of the duration that respondents had experienced the condition and may have been associated with life satisfaction. Furthermore, the numbers for some of those conditions were quite small despite the large sample size overall (e.g. 239 respondents had stroke and 371 respondents had cancer). The SYC data set was large and found to be quite representative in measurable terms to the UK population, but it was based only on a response rate at 17.8%. Furthermore, as the SYC data set was a single wave data set, we can only interpret our results as an association between life satisfaction and both the EQ-5D and long-standing health conditions.

Therefore, for further research, in order to establish a causality relationship between life satisfaction and both the EQ-5D and long-standing health conditions, additional research using longitudinal data sets is required to observe whether the association remains over time. Furthermore, it would improve the results to have clinical diagnoses confirmed for the conditions.

It is also unclear whether our results generalise to other measures of SWB measures or other GPBMs of health. Hence, further research should be undertaken to verify whether inclusion of any significant long-standing health condition as a unique dimension of health beyond the EQ-5D has a consistent effect on other SWB measures (e.g. health satisfaction, happiness and day effect) if data sets allow. It will also be useful to extend the work to the new five-level version of EQ-5D.

## Conclusion

Our study examined whether long-standing health conditions have a significant impact on life satisfaction alongside the EQ-5D. Our findings started to fill the gap in this hardly acknowledged research area using the SWB approach. Some long-standing health conditions were significant some of the time, but most were shown to have little relationship to life satisfaction when the EQ-5D dimensions were included in the models. Overall, none of long-standing health conditions was found to have a consistent impact on life satisfaction. However, more research is needed using clinically confirmed diagnoses and measures of severity.

## Abbreviations

GPBMs: Generic preference-based measures; SWB: Subjective well-being; BHPS: British household panel survey; SYC: South Yorkshire Cohort; s.d: Standard deviation.

## Competing interests

The authors declare that they have no competing interests.

## Authors’ contributions

JB conceived the research question and provided technical expertise for the study. MW undertook the data analysis and wrote the manuscript. All authors contributed to the writing of the manuscript and read and approved the final manuscript.

## Supplementary Material

Additional file 1: Table S1 Variables used in analyses. Click here for file
