# Supplementary material for: Examining the incremental impact of long-standing health conditions on subjective well-being alongside the EQ-5D
Source: Health Qual Life Outcomes. 2014 Apr 29;12:61. doi: 10.1186/1477-7525-12-61 (PMC4021593; doi:10.1186/1477-7525-12-61)
Supplement: Additional file 1: Table S1 — Variables used in analyses. [file 1477-7525-12-61-S1.docx]

Additional file 1. Table S1 Variables used in analyses

| Variables | Definitions |
| --- | --- |
| *Dependent variables* |  |
| Life satisfaction | How satisfied are you with your life as a whole?  (1-6 scale: Completely dissatisfied-completely satisfied) |
| *Independent variables* |  |
| EQ-5D dimensions |  |
| Mobility | =1 for level 2 |
| Self-care | =1 for level 2 |
| Usual activities | =1 for levels 2 and 3 |
| Pain/discomfort | =1 for levels 2 and 3 |
| Anxiety/depression | =1 for levels 2 and 3  *=0 reference category: no problems in each dimension* |
| Long-standing health conditions |  |
| Insomnia | =1 if yes |
| Diabetes | =1 if yes |
| Breathing problems | =1 if yes |
| High blood pressure | =1 if yes |
| Heart disease | =1 if yes |
| Osteoarthritis | =1 if yes |
| Stroke | =1 if yes |
| Cancer | =1 if yes  *=0 reference category: no* |
| Socio-demographic characteristics |  |
| Age, Age^2^ | =age, age squared |
| Gender | =1 if male |
| Ethnicity | =1 if white *reference category: non-white* |
| Educational attainment |  |
| GCSEs | =1 if yes *reference category: no qualification* |
| A levels | =1 if yes *reference category: below A levels* |
| Degree | =1 if yes *reference category: below degree* |
| Socio-economic status |  |
| White collar | =1 if yes *reference category: blue collar* |
| Current employment status | =1 if yes |
